# Subcutaneous MG1113 in severe hemophilia A and B: phase 1b study for safety, pharmacokinetics, and pharmacodynamics

**DOI:** 10.1016/j.rpth.2026.106910

**Published:** 2026-08-15

**Authors:** Chur-Woo You, Hee Jo Baek, Young-Shil Park, Bo Ram Kim, Hayoung Lee, Seon Hui Kim, Sung Ho Hwang, Eun Jin Choi

**Affiliations:** 1 Department of Pediatrics, Eulji University School of Medicine, Seoul, Republic of Korea; 2 Department of Pediatrics, Chonnam National University Hwasun Hospital, Chonnam National University Medical School, Gwangju, Republic of Korea; 3 Department of Pediatrics, Kyung Hee University Hospital at Gangdong, Seoul, Republic of Korea; 4 Medical Division, GC Biopharma, Yongin, Republic of Korea; 5 Department of Pediatrics, Daegu Catholic University School of Medicine, Daegu, Republic of Korea

**Keywords:** efficacy, hemophilia, pharmacodynamics, pharmacokinetics, safety, tissue factor pathway

## Abstract

**Background:**

MG1113 is an immunoglobulin G4 monoclonal antibody targeting the Kunitz-2 domain of tissue factor pathway inhibitor (TFPI), developed as a hemostatic rebalancing agent for patients with hemophilia A or B.

**Objectives:**

We aimed to investigate the safety, pharmacokinetics (PK), pharmacodynamics, and preliminary hemostatic effects of MG1113.

**Methods:**

This phase 1b clinical trial was conducted in a multicenter, prospective, stepwise dose-escalating (2.0, 3.0, and 3.3 mg/kg) design. MG1113 was administered subcutaneously once weekly for 8 weeks.

**Results:**

Fifteen male patients (*n* = 5/cohort) diagnosed with severe hemophilia A (*n* = 14) or B (*n* = 1) without inhibitors were enrolled. MG1113 was well tolerated, with an adverse event (AE) rate of 40.0% (6/15). No serious AEs, early withdrawals, or dose interruptions occurred. PK analysis demonstrated a nonlinear profile, with the peak plasma concentration at 33 to 48 hours post dose. Free TFPI and diluted prothrombin time (PT) decreased from baseline, while thrombin generation markers increased. Exploratory analysis showed a notable numerical reduction in annualized total bleeding rates at higher doses: 91.7% at 3.0 mg/kg and 76.2% at 3.3 mg/kg. The zero bleeding rate was 20.0%, 60.0%, and 40.0% at doses of 2.0, 3.0, and 3.3 mg/kg, respectively.

**Conclusion:**

MG1113, administered subcutaneously as a prophylaxis in patients with severe hemophilia A or B without inhibitors for 8 weeks, demonstrated an acceptable safety profile with a PK profile suitable for once-a-week dosing. The pharmacodynamics marker changes were consistent with TFPI inhibition, which was associated with reductions in bleeding events.

**Trial registration:**

This trial was prospectively registered at www.ClinicalTrials.gov (#NCT05493631).

## Essentials

• MG1113 is an antitissue factor pathway inhibitor, currently under clinical development.
• Fifteen people with severe hemophilia received MG1113 for 8 weeks.
• MG1113 showed a characteristic drug profile suitable for a once-a-week subcutaneous injection.
• MG1113 was well tolerated and demonstrated its potential as a rebalancing agent.

## 1 Introduction

Hemophilia is a rare, life-threatening, X-linked congenital disease caused by a deficiency or absence of factor (F)VIII (type A) or FIX (type B). According to the Report on the Annual Global Survey 2024 conducted by the World Federation of Hemophilia, a total of 271,918 individuals with hemophilia were identified from 135 countries; hemophilia A constituted 82.5% of the registered cases [1]. Despite the advances in factor replacement therapy, there are still unmet needs for this patient population, such as difficulties with treatment adherence, the inconveniences of frequent intravenous infusions, and inhibitor formation. The development of nonfactor therapies has transformed the landscape of hemophilia treatment by introducing subcutaneous injections with reduced frequency and novel targets [2]. Among them, the monoclonal antibodies targeting tissue factor pathway inhibitor (TFPI), the natural anticoagulant protein, are emerging as an alternative therapeutic modality. As of January 2026, there are two commercially available anti-TFPI inhibitors, concizumab and marstacimab, initially approved in 2023 and 2024, respectively [3,4]. Both products have demonstrated clinical benefits in terms of reduced annualized bleeding rates (ABRs), improved quality of life, and favorable safety profiles [5,6]. MG1113 is another anti-TFPI immunoglobulin G4 monoclonal antibody, currently under clinical development. MG1113 possesses a unique paratope that targets the Kunitz-2 (K2) domain of TFPI, with a high specificity for the Arg107 residue that plays a crucial role in the interaction between TFPI and the FXa active site [[7], [8], [9]]. Furthermore, the binding affinities of these agents vary significantly, which may influence their therapeutic windows and dosing regimens. MG1113 exhibits a potent binding affinity with a dissociation constant (*K*_D_) of approximately 46.65 pM [8]. This is comparable with concizumab (*K*_D_ = 25 pM) [10] and is more potent than marstacimab (*K*_D_ = 3.7-4.4 nM) [11]. Befovacimab (Bayer, BAY-1093884), another TFPI agent, has been reported to have an even higher affinity with a *K*_D_ of 10 pM [12]. The TFPI-neutralizing effects of MG1113 have been demonstrated in an *in vitro* setting using plasma of individuals with hemophilia as well as in an animal study using rabbits and cynomolgus monkeys [8,13]. The first-in-human (FIH) trial was conducted in both healthy volunteers and individuals with hemophilia A or B in a placebo-controlled, sequential dose escalation, phase 1 study (NCT03855696). The initial human subcutaneous dose of MG1113 applied in the aforementioned trial was 0.5 mg/kg/week, which was based on a conservative approach to set a lower dose than the human-equivalent doses that have no observed adverse events (AEs) (19.2 mg/kg multiplied by a safety factor of 10) and minimal pharmacologic activity (0.624 mg/kg). A pharmacokinetic (PK)/pharmacodynamic (PD) modeling using the FIH trial data provided a rationale to choose 2.0 mg/kg/week as a starting dose in the present trial, which would provide <50% inhibition of free TFPI. The optimal dose was presumed to be 3.0 mg/kg/week, which was simulated to produce 50% to 90% inhibition of free TFPI. The maximum weekly dose was set at 3.3 mg/kg, which was within the dose range tested in the FIH trial.

The main purpose of the present trial was to investigate the safety and tolerability of MG1113, along with its PK–PD profiles. The clinical efficacy of MG1113 was also examined, providing proof of concept for future trials.

## 2 Methods

### 2.1 Study design and patients

The trial was designed as a prospective, stepwise dose-escalating (three dose levels), phase 1b study and conducted at four sites in the Republic of Korea from August 2022 to December 2024. Male patients diagnosed with severe hemophilia A or B (FVIII or FIX level <1%) with or without inhibitors, aged between 19 and 60 years (inclusive), were eligible to participate in the trial if their body weight was ≥50 kg and their body mass index was between 18.5 and 29.9 kg/m^2^. Patients with congenital or acquired coagulation disorders other than hemophilia A or B were excluded from the trial. Other major exclusion criteria were as follows: personal or family history of thromboembolic events, coronary artery disease, or underlying risk factors for cardiovascular disease, including uncontrolled hypertension or diabetes mellitus; alanine aminotransferase or aspartate aminotransferase >3 times the upper limit of normal (ULN); hemoglobin <9.0 g/dL; absolute neutrophil count <1500/μL; platelet count <10^5^/μL; total bilirubin >2 mg/mL; serum creatinine >1.5 times the ULN; infections with hepatitis B virus, hepatitis C virus, or human immunodeficiency virus; use of emicizumab or immune tolerance induction therapy within 30 days before the screening; or the current use of systemic immunomodulators, such as high-dose systemic corticosteroid for ≥14 days, intravenous immunoglobulins, or rituximab. Prior to the first dose of MG1113, all eligible patients were required to undergo a 72-hour washout period for FVIII or a 96-hour washout period for FIX or any bypassing agents, whichever was applicable.

### 2.2 Ethical considerations

The trial was conducted according to the ethical principles of the Declaration of Helsinki, Good Clinical Practice, and relevant regulatory guidelines. All participants provided written informed consent prior to screening for the trial. The trial was prospectively registered in ClinicalTrials.gov (identifier: NCT05493631).

### 2.3 Intervention

**Figure 1:**
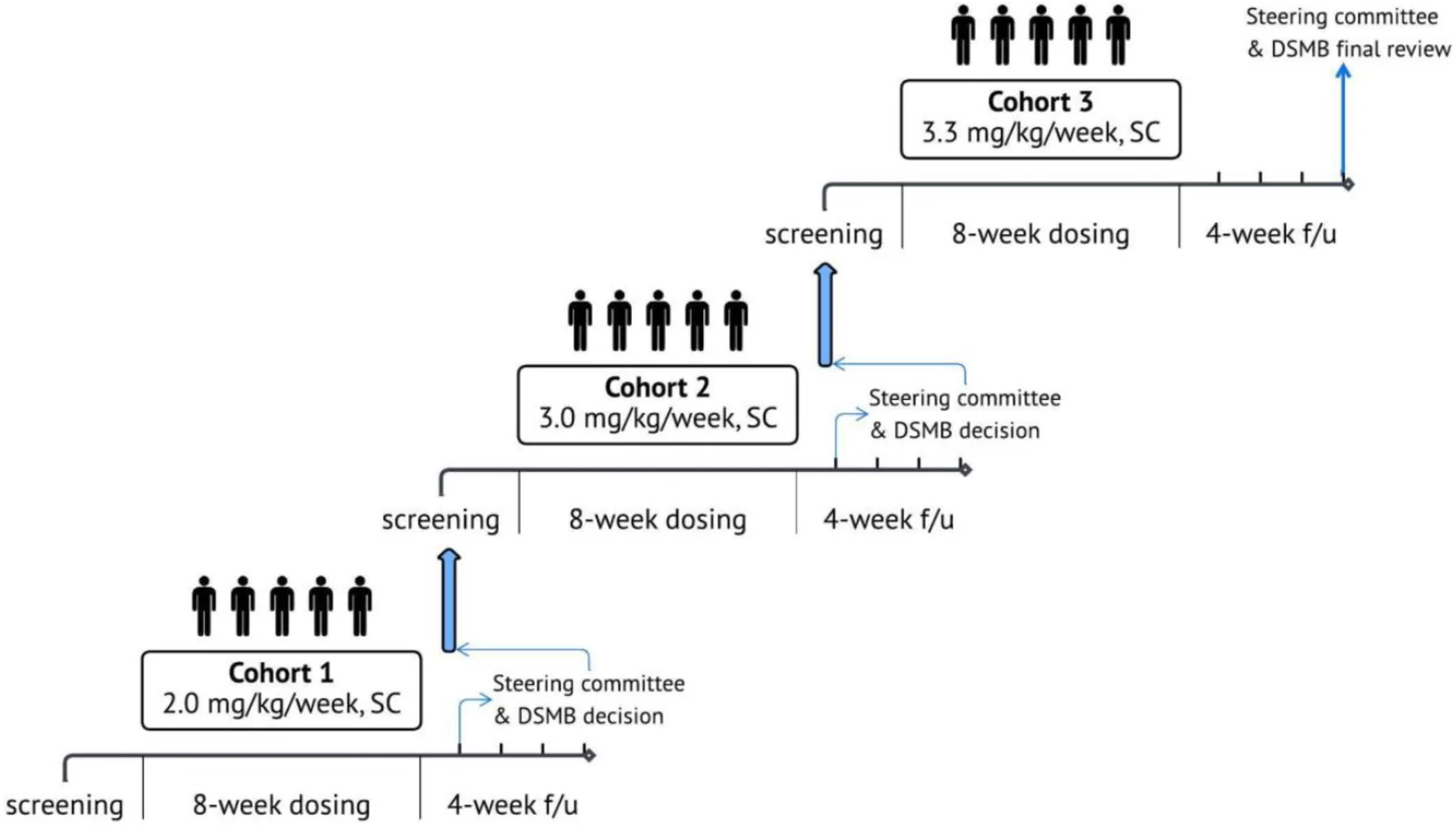
Study scheme. DSMB, Data and Safety Monitoring Board; F/U, follow-up; SC, subcutaneous injection.

MG1113 was administered subcutaneously by investigators or assigned medical practitioners (injection site: thigh, abdomen, or upper arm regions) once weekly for 8 weeks. The tested doses of MG1113, in sequential order, were 2.0 mg/kg (cohort 1), 3.0 mg/kg (cohort 2), and 3.3 mg/kg (cohort 3) (Figure 1). In cases of acute bleeding episodes, a previous prophylactic treatment could be used as a concomitant therapy, except for emicizumab; for those previously on emicizumab treatment, recombinant activated FVII was chosen for the case. Also, activated prothrombin complex concentrate was not used as a first-line treatment for acute bleeding but as a second-line option. Considering the additive effect of the additional hemostatic agent while receiving MG1113, the lowest dose of the concomitant replacement or bypassing therapy was applied (eg, FVIII ≤ 25 IU/kg or recombinant activated FVII ≤ 90 μg/kg). Meanwhile, no modification was made to the dosing of MG1113.

The steering committee and Data and Safety Monitoring Board, consisting of the investigators and five independent medical experts, reviewed each cohort’s data collected up to 1 week after the last dose, and a subsequent dose escalation was conducted only if no more than two patients experienced any of the following events in each cohort: an occurrence of a serious adverse drug reaction, an adverse drug reaction (ADR) of grade 3 or severe, or a thrombosis-related ADR; platelet count <10^5^/μL; serum creatinine >1.5 times the ULN; cardiac troponin I or cardiac troponin T > 5 times the ULN; fibrinogen less than half the lower limit of normal; a drug-induced liver injury; or PR interval >300 ms, QRS interval >150 ms, or QTc interval >500 ms from 12-lead electrocardiography. A drug-induced liver injury was defined if all three of the following conditions were met: (1) alanine aminotransferase or aspartate aminotransferase >3 times the ULN; (2) alkaline phosphatase less than twice the ULN, with total bilirubin more than twice the ULN; and (3) no clinically relevant reasons found to explain the two aforementioned conditions.

### 2.4 Procedure

Participants were hospitalized for 48 hours on the day of the first dose (day 1) and visited the study site weekly thereafter, up to 4 weeks after the last dose. At every visit, the following examinations and tests were conducted: physical examinations, vital signs, 12-lead electrocardiography, clinical laboratory tests, coagulation panel tests, measurement of FVIII and FIX activity, injection site examinations, recording of bleeding events, and assessment of AEs.

Serial blood collections for PK and PD analyses were performed on day 1 at the following time points: pre dose (0 hours) and 0.5, 1, 2, 4, 8, 12, 24, and 48 hours post dose. At subsequent visits, a predose blood sample was collected at each visit, as well as at 48 hours post dose after the fifth dose and at 24, 48, and 72 hours post dose after the eighth dose. PD markers included free plasma TFPI, diluted PT, residual TFPI activity, D-dimer, fibrinogen, and prothrombin fragment 1+2, and parameters from the thrombin generation assay (TGA), such as lag time for thrombin generation, peak thrombin generation (peak height), and endogenous thrombin potential (ETP).

Blood samples for the antidrug antibody (ADA) assay were collected right before the first dose and at weeks 4 and 8. Measurement of ADA, free TFPI, and residual TFPI activity was performed at the Korea Institute of Toxicology. TGA and analysis of plasma drug concentration, diluted PT, and prothrombin fragment 1+2 were performed at Medpace Bioanalytical Laboratories. The remaining laboratory tests were performed at Seoul Clinical Laboratories. The plasma concentration of MG1113 was determined using a ligand-binding assay. ADA was measured using the enzyme-linked immunosorbent assay in a stepwise approach consisting of screening, confirmation by immunodepletion, and titer determination.

### 2.5 Outcomes

The safety of MG1113 was assessed through evaluation of AEs, ADRs, serious AEs, AEs of special interest, injection site AEs, physical examination, 12-lead electrocardiography, vital signs, and clinical laboratory tests, including ADA. An AE of special interest was defined as an AE related to thrombosis, regardless of its severity. The severity of an AE was graded according to the criteria presented in the Common Terminology Criteria for Adverse Events, version 5.0.

The following PK parameters after a single dose and at steady state were calculated: area under the plasma concentration-time curve from time of dosing to the last measurable concentration (AUC_last_), area under the curve (AUC) from time zero extrapolated to infinity (AUC_inf_), AUC within a dosing interval at steady state (AUC_tau_), maximum plasma drug concentration (*C*_max_), *C*_max_ at steady state (*C*_max,ss_), minimum drug concentration at steady state (*C*_min,ss_), time to reach *C*_max_ (*T*_max_), *T*_max_ at steady state (*T*_max,ss_), elimination half-life (*t*_1/2_), apparent drug clearance from plasma after subcutaneous administration (CL/F), CL/F at steady state (CL_ss_/F), apparent volume of distribution after subcutaneous administration (Vd/F), Vd/F at steady state (Vd_ss_/F), and accumulation index.

For the PD markers, maximum observed response value (*E*_max_), time to *E*_max_ (*T*_max_), and area under the effect curve (AUEC) were calculated.

The efficacy of MG1113 was evaluated through the proportions of patients experiencing any bleeding episodes, total bleeding episodes ≤1, or zero bleeding episodes; ABR and annualized spontaneous bleeding rate; and the severity of bleeding.

### 2.6 Statistical analysis

No formal sample size calculation was performed, as no hypothesis testing was planned. Five patients were targeted for enrollment in each cohort.

Baseline characteristics, AEs, PK–PD parameters, and efficacy parameters were summarized using descriptive statistics. AEs were coded using MedDRA version 27.0.

The dose proportionality of PK parameters (*C*_max_, AUC_last_, and AUC_tau,ss_) and PD parameters (*E*_max_ and AUEC) was evaluated using the Kruskal–Wallis test for dose-normalized values as well as using a power model (In(parameter) = *β*0 + *β*1·ln(dose)). For the latter, dose proportionality was concluded if the 90% CI of *β*1 was within the predefined acceptance interval of 0.55 to 1.45.

Any PK data below the lower limit of quantitation (LLOQ) prior to the first dose or *T*_max_ were imputed as 0, and all other missing values, values below LLOQ, and values above the upper limit of quantitation (ULOQ) were not imputed and were treated as blank data. For the PD parameters, any missing, below LLOQ, or above ULOQ values were treated as blank data unless >5% of the data were affected, in which case the following replacement rule was applied: 0 for missing, LLOQ for below LLOQ, or ULOQ above ULOQ. No missing data were imputed for the safety and efficacy analyses.

The PK–PD relationship was evaluated using a linear regression. For ABR analysis, the mean and its 80% CI were estimated using a negative binomial model. As a post hoc analysis, an exploratory exposure–response graph was plotted using plasma drug concentration and percent reduction in free anti-TFPI levels from baseline, while individual bleeding events were overlaid. From this, a drug concentration threshold was proposed as an effective concentration.

All statistical analyses were conducted using SAS version 9.4 (SAS Institute). PK and PD parameters were calculated by noncompartmental analysis using the NonCompart package in R version 4.3.0. Unless otherwise specified, the significance level was set at 5%.

## 3 Results

### 3.1 Patients

A total of 15 male patients with severe hemophilia were registered as planned (5 per cohort), all received the eight weekly subcutaneous injections at the assigned dose, and all were included in the analyses. Age ranged from 23 to 54 years, with medians of 45, 27, and 26 years in cohorts 1, 2, and 3, respectively (Table 1). One patient in cohort 2 had hemophilia B; the remaining (*n* = 14) were diagnosed with hemophilia A, and none of the patients had inhibitors. The duration of hemophilia was similar to the patient’s age, as most of them were diagnosed early in their life. All patients were on factor replacement therapy, mostly (14/15) with a prophylaxis regimen (Table 1). The average number of total bleeding episodes per month within 24 weeks prior to the study ranged from 0 to 8, with medians of 2, 2, and 5 in cohorts 1, 2, and 3, respectively.

**Table 1:** Demographic and baseline characteristics.

	Cohort 1 2.0 mg/kg QW (*n* = 5)	Cohort 2 3.0 mg/kg QW (*n* = 5)	Cohort 3 3.3 mg/kg QW (*n* = 5)	All patients (*N* = 15)
**Sex, *n* (%)**				
Male, *n* (%)	5 (100.0)	5 (100.0)	5 (100.0)	15 (100.0)
**Age, *y***				
Median (range)	45.0 (25.0-53.0)	27.0 (23.0-54.0)	26.0 (23.0-38.0)	32.0 (23.0-54.0)
**BMI, kg/m^2^**				
Median (range)	23.4 (19.1-29.2)	23.3 (19.6-24.9)	23.3 (20.0-26.9)	23.3 (19.1-29.2)
Race/ethnicity				
Asian/Korean (%)	100	100	100	100
**Hemophilia type, *n* (%)**				
Hemophilia A	5 (100.0)	4 (80.0)	5 (100.0)	14 (93.3)
Hemophilia B	0	1 (20.0)	0	1 (6.7)
**Duration of hemophilia, *y***				
Median (range)	40.7 (25.0-53.8)	22.3 (18.8-43.3)	26.3 (20.7-38.1)	26.3 (18.8-53.8)
**Bleeding history, *n* (%)**^a^				
Yes	5 (100.0)	5 (100.0)	4 (80.0)	14 (93.3)
No	0	0	1 (20.0)	1 (6.7)
**No. of bleeding events/month**^a^				
Median (range)	2.0 (1.0-3.0)	2.0 (2.0-4.0)	5.0 (0.0-8.0)	2.0 (0.0-8.0)
**Bleeding type, *n* (%)**				
Spontaneous bleeding	4 (80.0)	5 (100.0)	4 (100.0)	13 (92.9)
Traumatic bleeding	1 (20.0)	1 (20.0)	1 (25.0)	3 (21.4)
**Treatment, *n* (%)**				
Factor replacement therapy	5 (100.0)	5 (100.0)	5 (100.0)	15 (100.0)
Other	0	0	0	0
**Treatment regimen, *n* (%)**^b^				
Prophylaxis	5 (100.0)	5 (100.0)	4 (80.0)	14 (93.3)
On-demand	2 (40.0)	0	1 (20.0)	3 (20.0)

BMI, body mass index.

a Data within 24 weeks prior to the study.

b Duplicate counting.

### 3.2 Safety

AEs were observed in 2, 1, and 3 patients in cohorts 1, 2, and 3, respectively, which resulted in an overall incidence rate of 40.0% (6/15) (Table 2). All AEs were grade 1 or 2 in severity, except one event—blood creatine phosphokinase increased—observed in cohort 3. The most frequent AE was injection site reaction, with six events experienced by one patient in each cohort (20.0%). All of the injection site reactions were grade 1 in severity, resolved without treatment, and were assessed as ADRs. No other ADR was identified, and no systemic pruritus or urticaria was observed. All the subcutaneous injections were made in the abdomen, except for one occasion in cohort 3, where the injection was made both in the abdomen and in the arm at the same time. No serious AEs or AEs of interest were reported, and no AEs resulted in early withdrawals or dose interruptions. None of the patients developed ADA throughout the study period.

**Table 2:** Summary of adverse events.

	Cohort 1 2.0 mg/kg QW (*n* = 5)	Cohort 2 3.0 mg/kg QW (*n* = 5)	Cohort 3 3.3 mg/kg QW (*n* = 5)	All patients (*N* = 15)
**Any adverse events**	**2 (40.0)**	**1 (20.0)**	**3 (60.0)**	**6 (40.0)**
Injection site reaction^a^	1 (20.0)	1 (20.0)	1 (20.0)	3 (20.0)
Catheter site hypoesthesia	1 (20.0)	0	0	1 (6.7)
Pyrexia	0	0	1 (20.0)	1 (6.7)
Abdominal pain	1 (20.0)	0	0	1 (6.7)
Enteritis	0	0	1 (20.0)	1 (6.7)
Gastritis	1 (20.0)	0	0	1 (6.7)
Stomatitis	1 (20.0)	0	0	1 (6.7)
Vomiting	1 (20.0)	0	0	1 (6.7)
Laryngeal inflammation	1 (20.0)	0	0	1 (6.7)
Oropharyngeal pain	0	0	1 (20.0)	1 (6.7)
Productive cough	0	0	1 (20.0)	1 (6.7)
Rhinorrhea	1 (20.0)	0	0	1 (6.7)
ALT increased	0	0	1 (20.0)	1 (6.7)
AST increased	0	0	1 (20.0)	1 (6.7)
Blood CPK increased	0	0	1 (20.0)	1 (6.7)
Nasopharyngitis	0	0	1 (20.0)	1 (6.7)
Myalgia	0	0	1 (20.0)	1 (6.7)
Headache	0	0	1 (20.0)	1 (6.7)

MedDRA terminology was modified to American spelling.

ALT, alanine aminotransferase; AST, aspartate aminotransferase; CPK, creatine phosphokinase.

a Assessed as an adverse drug reaction.

### 3.3 Pharmacokinetics

**Figure 2:**
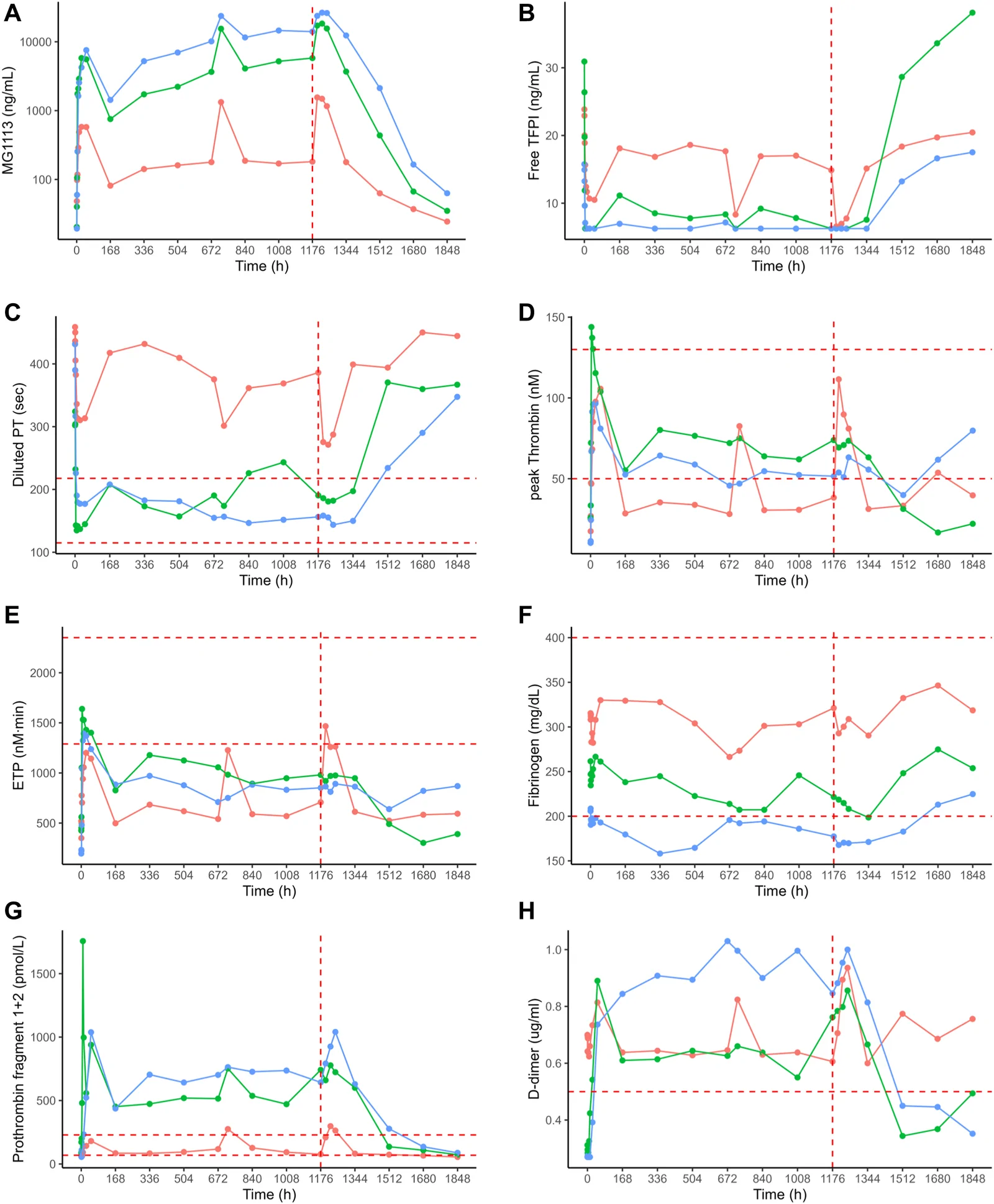
Mean plot of MG1113 plasma concentration and pharmacodynamic markers. (A) Plasma concentration of MG1113 over time on a semilog scale. (B–H) Plasma levels of pharmacodynamic markers. Cohorts are color-coded as follows: red for cohort 1, green for cohort 2, and blue for cohort 3. The red-dashed vertical line denotes the time point of the last MG1113 dose, that is, at the start of the eighth week. The red-dashed horizontal line(s) represent the normal ranges, if applicable. ETP, endogenous thrombin potential; PT, prothrombin time; TFPI, tissue factor pathway inhibitor.

The plasma concentration of MG1113 over time is displayed in Figure 2A, and single-dose and steady-state PK parameters are summarized in Supplementary Tables S1 and S2, respectively. After a single subcutaneous dose, MG1113 was absorbed slowly (mean *T*_max_, 43-92 hours), and exposure increased disproportionately between 2.0 mg/kg (cohort 1) and 3.0 mg/kg (cohort 2), demonstrating a nonlinear PK profile. *C*_max_ and AUC_last_ increased from 2.0 to 3.0 mg/kg by 10.7-fold and 9.4-fold, respectively, whereas the fold increases of these parameters from 3.0 to 3.3 mg/kg were 1.2-fold for both. At 2.0 mg/kg, a high intersubject variability was observed (coefficient of variation [CV] > 100%).

At steady state, systemic exposure profoundly increased, with a greater accumulation tendency at the higher dose: the accumulation index in cohorts 1, 2, and 3 was 3.68, 3.87, and 5.49, respectively. The mean (CV%) *C*_max,ss_ (ng/mL) was 1,570 (118), 18,638 (52), and 27,520 (46) in cohorts 1, 2, and 3, respectively. The intersubject variability for *C*_max,ss_ and AUC_tau_ remained high in cohort 1 (CV > 100%). The mean *T*_max,ss_ ranged from 33 to 48 hours; compared with *T*_max_ after the single dose, *T*_max,ss_ decreased substantially in cohort 1, while it remained at a similar level in cohorts 2 and 3. With increasing doses, the clearance (CL_ss_/F) decreased, *t*_1/2_ shortened, and volume of distribution markedly decreased (Supplementary Table 2). The plasma concentration of MG1113 returned to baseline levels at 2 to 3 weeks after the last dose.

Dose proportionality was not demonstrated either by the Kruskal–Wallis test or by the power model (90% CIs of the slope estimate ranged from 4.49 to 8.85 at single dose and from 5.08 to 8.58 at steady state) (Supplementary Table S3).

### 3.4 Pharmacodynamics

From the analysis of PD parameters, a decreasing trend from baseline was observed in free TFPI (Figure 2B) and diluted PT (Figure 2C). At steady state, the reductions in residual TFPI activity (*E*_max_, −0.34 to −0.10 unit/mL) and lag time for thrombin generation (*E*_max_, −2.79 to −1.73 minutes) were modest, with great intersubject variability. Meanwhile, dose-dependent increasing trends were observed with peak thrombin generation, ETP, D-dimer, and prothrombin fragment 1+2 (Figure 2D–H). Fibrinogen was generally maintained at a stable level (Figure 2F).

The dose proportionality tests across multiple PD markers yielded mixed results depending on the statistical methods. Based on the Kruskal–Wallis test, most markers, except for D-dimer and prothrombin fragment 1+2, demonstrated significant differences in dose-normalized E_max_ and AUEC, suggesting nonproportionality. The power model also revealed nonproportionality or inconclusive results, except for the AUEC of free TFPI, which met the proportionality criteria with a 90% CI of −1.26 to −0.78. Except for D-dimer, *E*_max_ curves of most PD parameters showed dose-dependent reductions (free TFPI, diluted PT, peak thrombin generation, and fibrinogen) or an increase (prothrombin fragment 1+2) (Supplementary Figure S1).

**Figure 3:**
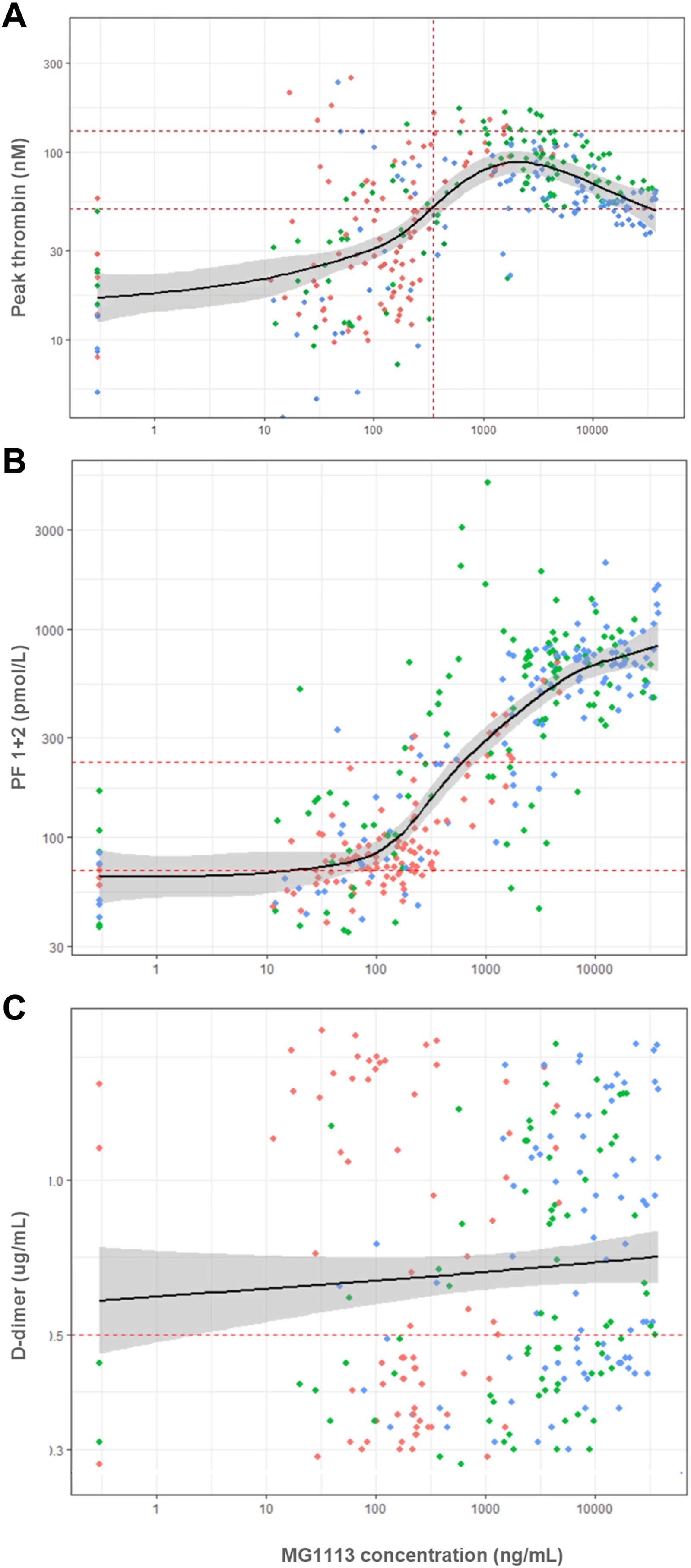
Correlation between pharmacodynamic markers and MG1113 concentration. Correlation plots for (A) peak thrombin, (B) prothrombin fragment 1+2 (PF 1+2), and (C) D-dimer. Cohorts are color-coded as follows: red for cohort 1, green for cohort 2, and blue for cohort 3. The red-dashed horizontal line(s) represent the normal ranges of each parameter.

The linear regression analysis for the PK–PD relationship revealed a weak statistical association, with *R*^2^ values ranging from 0.000822 to 0.222, suggesting that drug exposure alone may not fully account for the observed PD variability. However, exploratory visual inspection of the plots suggested a potential dose-dependent trend in certain thrombin generation parameters within the low- to mid-dose ranges. Notably, this trend did not persist at the highest dose, where an unexpected decrease was observed. This observation may be attributed to a potential “hook effect” or other compensatory mechanisms, warranting further investigation in future studies (Figure 3A). The same trend was also observed with ETP. Unlike the TGA results, prothrombin fragment 1+2 levels exhibited a dose-dependent increase across all dose ranges, and most of the patients in cohort 3 had fragment 1+2 levels above the normal range (Figure 3B). Meanwhile, D-dimer concentrations were scattered below or above the upper limit (0.5 μg/mL) of normal without an obvious relationship with MG1113 concentration (Figure 3C).

### 3.5 Efficacy

New spontaneous or traumatic bleeding episodes occurred in nine patients (60.0%) across three cohorts, with a total of 23 events. Specifically, there were 12 total bleeding episodes in four (80.0%) patients in cohort 1, 2 episodes in two (40.0%) patients in cohort 2, and 9 episodes in three (60.0%) patients in cohort 3. Clotting factor (FVIII or FIX) replacement therapy was administered for all bleeding episodes in cohort 1 (12 episodes) and cohort 2 (2 episodes), and 4 out of 9 episodes in cohort 3 required replacement therapy. Of the five events that did not necessitate replacement therapy, three were associated with bleeding secondary to a prior history of hemorrhoids. No bypassing agents or emicizumab were used.

The number of patients experiencing ≤1 bleeding episode was 3 (60.0%), 5 (100.0%), and 3 (60.0%) in cohorts 1, 2, and 3, respectively, and the number of participants having zero bleeding episode was 1 (20.0%), 3 (60.0%), and 2 (40.0%) in cohorts 1, 2, and 3, respectively.

**Figure 4:**
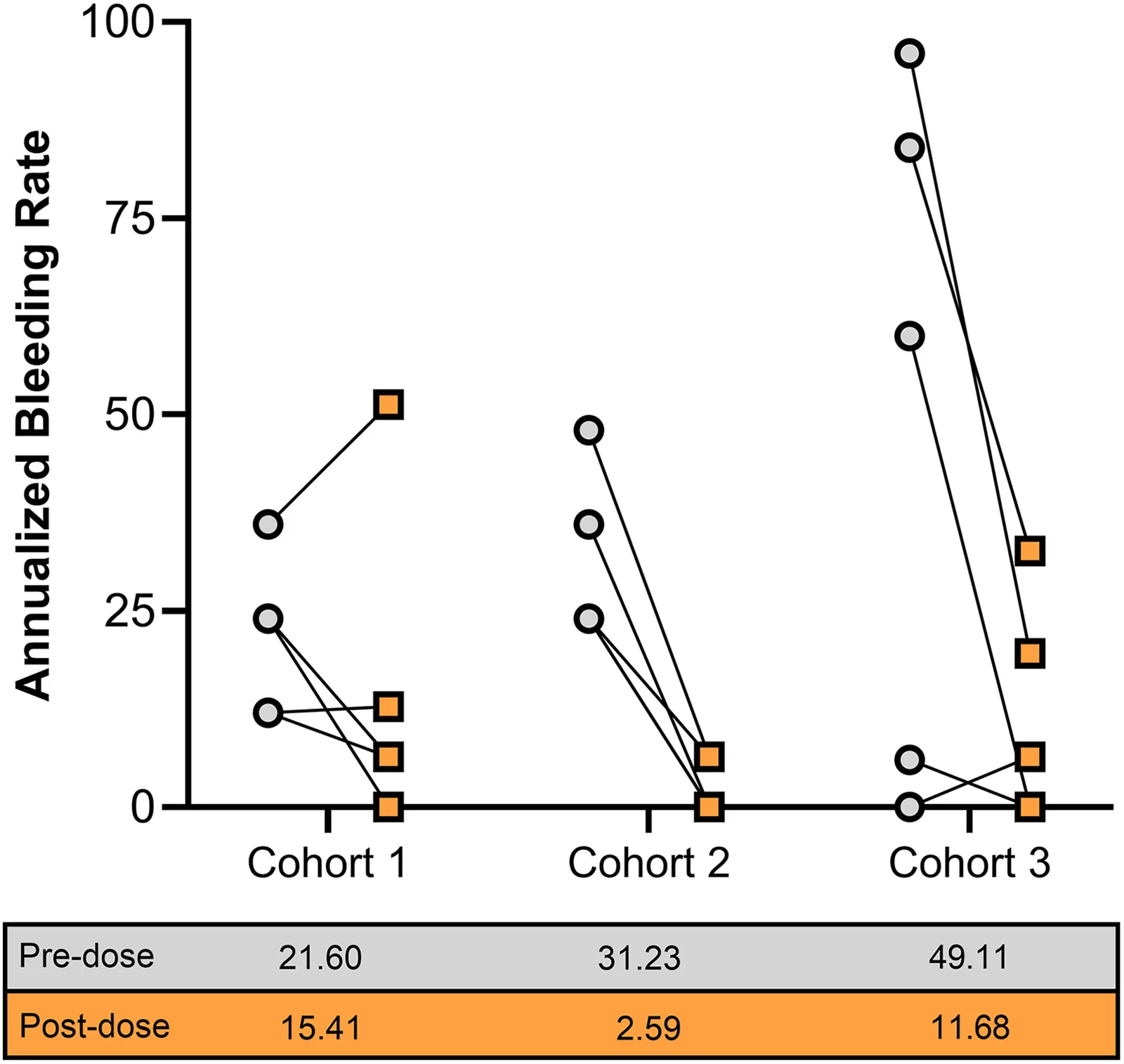
Estimated mean annualized bleeding rates (ABRs) for total bleeding at pre and post dosing. The total ABR, encompassing both spontaneous and traumatic bleeding, was calculated using a negative binomial model. For each cohort, individual trajectories for total ABRs are illustrated. When multiple patients exhibited the same ABRs, data points are superimposed. The mean ABR at pre dose and post dose for each cohort are presented in the table below the graph.

The estimated total ABR from the negative binomial model indicated a decrease in total bleed in all three cohorts (Figure 4). The extent of total ABR reduction after treatment was greater in cohorts 2 and 3 (91.7% reduction, *P* < .0001 and 76.2% reduction, *P* < .0001, respectively) compared with that observed in cohort 1 (28.7% reduction, *P* = .4296). The reductions in annualized spontaneous bleeding rate were also statistically significant in cohorts 2 and 3, with a 95.8% reduction (*P* < .0001) and an 86.8% reduction (*P* = .0029), respectively.

During treatment with MG1113, the frequency and severity of bleeding were lower and milder than during the posttreatment follow-up period. During treatment, 78.3% (18/23) of the events were mild (the rest were moderate), whereas during the posttreatment 4-week follow-up period, 47.1% (16/34) were mild and 52.9% (18/34) were moderate.

An exploratory post hoc analysis identified 300 ng/mL as a potential threshold for optimal PD response and clinical efficacy. While not prespecified, this value provides a hypothesis-generating basis for dose selection in future confirmatory studies. The graph also showed that a 75% reduction in free TFPI activity from baseline was related to no bleeding, which was primarily observed in cohort 2 (Supplementary Figure S2).

## 4 Discussion

For half a century, factor replacement therapy has served as a cornerstone of hemophilia management. Although maintaining the coagulation factor trough levels above 1% has been associated with a reduction in bleeding episodes, contemporary treatment paradigms have shifted toward higher target trough levels (≥3%-5%), tailored to individual patient needs, thereby substantially increasing treatment burden [14,15]. In addition, the relatively high incidence of inhibitor development and challenges in maintaining good treatment compliance have been recurring issues with the conventional factor replacement strategies. The introduction of nonfactor treatments, such as factor mimetics or rebalancing agents, has expanded the treatment options to patients with hemophilia [16]. Anti-TFPI antibodies, as a rebalancing agent, restore hemostasis by inhibiting TFPI, the natural coagulant protein. Unlike emicizumab, which mimics FVIIIa, anti-TFPI does not target specific missing factors; therefore, it can be applied to both patients with hemophilia A and B. In theory, the inhibitor status may not affect the mechanism of action of anti-TFPI, which is as promising as it could be. However, anti-TFPI antibodies harbor possible drawbacks as well. Specifically, anti-TFPI agents may overcorrect the bleeding pathway and result in thrombosis risk, which caused the early termination of befovacimab [17], a temporary hold of two phase 3 trials of concizumab [18], and the death of a patient treated with marstacimab for 3 years [19]. Recognizing these class-specific prothrombotic risks and historical safety challenges, the clinical protocol for MG1113 was designed with a highly conservative dose-escalation scheme and rigorous, proactive safety monitoring. This approach aims to carefully navigate the hypercoagulable potential inherent to the class while finding its distinct therapeutic place amid a shifting hemophilia treatment paradigm that includes FVIII mimetics and long-acting factor concentrates.

In this phase 1b trial conducted in 15 male patients with severe hemophilia A (*n* = 14) or B (*n* = 1), without inhibitors, MG1113 showed a favorable safety profile, with an overall incidence of AEs of 40%, which were mostly mild. The incidence of ADRs was low (20%), which solely included injection site reactions that were mild (grade 1) and self-resolving. This result is consistent with the class effect of subcutaneous biological agents. No serious AEs, thromboembolic events, or bleeding-related safety concerns were observed. Also, no patients were prematurely withdrawn from the trial or discontinued the treatment due to AEs. The most common (incidence rate ≥5%) ADRs listed on the product label of concizumab are injection site reactions and urticaria. For marstacimab, the most common (incidence rate ≥3%) ADRs include injection site reactions, headache, and pruritus [20]. In the present trial, no systemic pruritus or urticaria was observed. Moreover, no patients developed ADA during the entire trial period, including the 4-week posttreatment follow-up window. In early-phase to phase 3 trials of concizumab, the incidence of ADA varied from 0% to 26% [18,21,22]. In the case of marstacimab, the overall incidence of ADA formation was reported to be 11.5% from a meta-analysis [5]. However, the presence of ADA did not affect the efficacy of both agents, and its clinical significance has not been clearly elucidated. While no ADAs or clinical thromboembolic events were reported during this study, these findings are not reassuring of long-term safety. Given the small sample size and the limited duration of drug exposure, the potential for immunogenicity and prothrombotic risks cannot be fully ruled out. Therefore, continuous and rigorous safety monitoring in larger cohorts with extended treatment periods is essential in future clinical investigations.

The nonlinear PK profile with the long *T*_max_ of MG1113 attests to its suitability for infrequent dosing. After subcutaneous administration, MG1113 was slowly absorbed, with a prolonged *T*_max_ (43-92 hours) and substantial individual variability, particularly at the low dose (2.0 mg/kg). Exposure increased disproportionately between 2.0 and 3.0 mg/kg, consistent with the nonlinear, target-mediated drug disposition (TMDD) [23], and the variability decreased at higher doses and with repeated administrations. The PK outcomes observed in the present trial were consistent with those previously simulated from the TMDD model of monkeys, although the model overestimated the exposure and underestimated the disproportionality between the doses [13]. The two approved anti-TFPI inhibitors—concizumab and marstacimab—have adopted a loading dose strategy to achieve rapid target saturation and to minimize PK–PD variability during the initial dosing period [5,6]. In the early-phase trials of concizumab in hemophilia A or B without inhibitors, no loading dose was given; however, this was recognized as a “weakness in the trial design,” and a loading dose was implemented in later trials [24]. As the present trial was in an early phase of development, the safety of the participants was prioritized over the theoretical dose regimen. However, the characterized PK profile of MG1113 suggests that a loading dose strategy could shorten the time to reach the optimal TFPI suppression and restoration of thrombin generation. It can be evaluated prospectively in future dose-finding studies.

The disproportionate increase in systemic exposure between 2.0 and 3.0 mg/kg cohorts was associated with nondose proportionality in PD parameters. Most PD effects, such as decreases in free TFPI and diluted PT and increases in thrombin generation parameters (peak height and ETP), D-dimer, and prothrombin fragment 1+2, were pronounced above 2.0 mg/kg and reached a plateau at ≥3.0 mg/kg. The concentration–effect relationship for free TFPI and peak thrombin generation demonstrated a hook effect, as an unexpected decrease was observed in both markers, primarily observed in cohort 2, despite similar drug exposure between cohorts 2 and 3. Prothrombin fragment 1+2 levels increased with the rising plasma drug concentration and exceeded the ULN in most cohort 3 participants. As a stable cleavage product released during the conversion of prothrombin to thrombin, prothrombin fragment 1+2 serves as a reliable *in vivo* marker of thrombin generation [25,26] and thromboembolic risk [[27], [28], [29]]. Although several participants exhibited marked elevations of prothrombin fragment 1+2 (>300 pmol/L) and D-dimer (>0.5 μg/mL), no thromboembolic event occurred during the present trial. Fibrinogen levels remained within the normal range, consistent with previous observations from the concizumab trials [6]. These changes in PD parameters in association with drug exposure indicate that MG1113 effectively suppresses TFPI activity, with downstream effects on coagulation markers. Although the hemostatic effects did not result in an uncontrolled increase in systemic fibrin turnover, the clear dose-dependent increase in procoagulant markers, specifically D-dimer and prothrombin fragment 1+2, particularly in cohort 3 (3.3 mg/kg), signals a state of heightened coagulation activation. Also, the observation that these markers rise in tandem with MG1113 concentration suggests a narrow therapeutic window. As stated earlier, the dual-domain anti-TFPI inhibitor befovacimab, which binds to both K1 and K2 domains, showed excessive procoagulant activity, and its clinical development program was discontinued [17]. K2-selective antibodies—concizumab and marstacimab—showed lower rates of thromboembolic events; however, warnings remaining on their labels and a recent death of a patient treated with marstacimab [19] accentuate the importance of close monitoring for potential thromboembolic events while using an anti-TFPI agent. Although MG1113 recognizes a unique epitope, its core inhibitory mechanism—targeting the Kunitz-2 domain to prevent FXa binding—aligns with the established pharmacologic class of anti-TFPI antibodies. Therefore, while its molecular binding profile is distinct, MG1113 shares the fundamental class-level effects on the coagulation cascade, and its clinical differentiation will need further validation in larger trials. Considering the class-wide risk of thrombosis and the dose-dependent fluctuations in D-dimer and prothrombin fragment 1+2 observed in the present trial, regular laboratory assessments to identify patients at risk of subclinical hypercoagulability must be implemented in future clinical development of MG1113.

Meanwhile, exploratory clinical trends were also observed, including a reduction in ABR for both total bleeding and spontaneous bleeding. All cohort 2 patients experienced ≤ 1 bleed during the trial, with 60% achieving zero bleeds. The exploratory post hoc analysis suggested that a potential therapeutic threshold (such as a 75% reduction in free TFPI activity from baseline) was associated with zero bleeding. Although these are encouraging results, the ABR reductions with MG1113 observed in this study appear relatively modest compared with those with the current standard treatments for hemophilia A, such as emicizumab and the recently approved efanesoctocog alfa. Clinical data for emicizumab have consistently shown median ABRs below 1.0 across various HAVEN trials, with a high proportion of patients achieving zero bleeds [30]. Similarly, efanesoctocog alfa, a high-sustained FVIII replacement therapy, reported an estimated median ABR of 0.71 in its pivotal XTEND-1 study, providing near-normal factor activity levels for a significant portion of the week [31]. Although the mechanism of “rebalancing” the coagulation cascade via TFPI inhibition might not achieve the same level of hemostatic peak as direct factor replacement or FVIII-mimetic agents, anti-TFPI agents hold a clinical advantage as a potential therapeutic alternative for patients with hemophilia B, mostly managed with intravenous injections of standard or extended half-life FIX concentrates. However, the inclusion of only a single participant with hemophilia B is entirely insufficient to draw definitive conclusions, rendering these data preliminary. A future clinical program of MG1113 should rigorously evaluate its relative utility compared with these established factors and nonfactor therapeutics in a larger cohort of patients with both hemophilia types.

Despite some limitations, such as small sample size, short follow-up, and baseline imbalances between cohorts, this phase 1b trial provides valuable proof-of-concept evidence that MG1113 can safely modulate TFPI activity and reduce bleeding risk, supporting its further evaluation in larger, controlled trials.

## 5 Conclusion

MG1113, administered subcutaneously as a prophylaxis in patients with severe hemophilia A or B without inhibitors, demonstrated a favorable safety profile with no observed evidence of immunogenicity, nonlinear PK consistent with TMDD, and PD marker changes characteristic of TFPI inhibition. A numerical reduction in ABRs suggests clinical benefit. These results provide a rationale for future clinical development, including exploration of optimized dosing strategies.

## Supplementary material

Supplementary Tables S1-S3 snd Figures S1-S2
