# Supplementary material for: Subcutaneous MG1113 in severe hemophilia A and B: phase 1b study for safety, pharmacokinetics, and pharmacodynamics
Source: Res Pract Thromb Haemost. 2026 Aug 15;10(6):106910. doi: 10.1016/j.rpth.2026.106910 (PMC13584034; doi:10.1016/j.rpth.2026.106910)
Supplement: Supplementary file 1 [file mmc1.docx]

**Supplemental Data**

**Supplemental Table 1.** **Descriptive statistics of single-dose pharmacokinetic parameters**

|  | **Cohort 1**  **2.0 mg/kg QW**  **(n=5)** | **Cohort 2**  **3.0 mg/kg QW**  **(n=5)** | **Cohort 3**  **3.3 mg/kg QW**  **(n=5)** |
| --- | --- | --- | --- |
| C_max_, ng/mL | 603.04 (120.71) | 6444.00 (45.41) | 7564.00 (53.76) |
| T_max_, h | 91.98 (78.46) | 43.07 (25.17) | 47.82 (0.57) |
| AUC_last_, h*ng/mL | 62102.24 (114.39) | 586240.80 (39.98) | 732507.35 (53.29) |
| AUC_inf_, h*ng/mL | NA | NA | NA |
| t_1/2_, h | NA | NA | NA |
| CL/F, mL/h/kg | NA | NA | NA |
| Vd/F, mL/kg | NA | NA | NA |

Data are mean (coefficient of variation %). The terminal phase parameters—AUC_inf_, t_1/2_, CL/F, and Vd/F—were not calculated, as the terminal slope could not be determined. AUC_last_, area under the plasma concentration-time curve (AUC) from time of dosing to the last measurable concentration; AUC_inf_, AUC from time zero extrapolated to infinity; CL/F, apparent drug clearance from plasma after subcutaneous administration; C_max_, maximum plasma drug concentration; QW, once per week; T_max_, time to reach C_max_; t_1/2_, elimination half-life; Vd/F, apparent volume of distribution after subcutaneous administration.

**Supplemental Table 2.** **Descriptive statistics of steady-state pharmacokinetic parameters**

|  | **Cohort 1**  **2.0 mg/kg QW**  **(n=5)** | **Cohort 2**  **3.0 mg/kg QW**  **(n=5)** | **Cohort 3**  **3.3 mg/kg QW**  **(n=5)** |
| --- | --- | --- | --- |
| C_max,ss_, ng/mL | 1570.00 (117.67) | 18638.00 (52.21) | 27520.00 (46.24) |
| C_min,ss_, ng/mL | 164.30 (32.85) | 3119.00 (51.68) | 11686.00 (59.20) |
| T_max,ss_, h | 32.99 (40.29) | 42.93 (24.98) | 47.65 (35.68) |
| AUC_tau,ss_, h*ng/mL | 155951.15 (106.35) | 2163614.10 (55.63) | 3769698.58 (50.28) |
| AUC_inf_, h*ng/mL | 196516.30 (83.50) | 2589931.79 (55.04) | 5204176.05 (53.26) |
| t_1/2_, h | 194.33 (36.38) | 124.83 (43.39) | 105.43 (47.62) |
| CL_ss_/F, mL/h/kg | 0.0277 (77.59) | 0.0017 (49.73) | 0.0013 (83.01) |
| Vd_ss_/F, mL/kg | 8.06 (81.82) | 0.34 (76.01) | 0.25 (130.94) |
| Accumulation index | 3.68 (61.97) | 3.87 (46.43) | 5.49 (49.02) |

Data are mean (coefficient of variation %). AUC_inf_, area under the plasma concentration-time curve (AUC) from time zero extrapolated to infinity; AUC_tau,ss_, AUC within a dosing interval at steady state; CL_ss_/F, apparent drug clearance from plasma after subcutaneous administration at steady state; C_max,ss_, maximum plasma drug concentration at steady state; C_min,ss_, minimum plasma drug concentration at steady state; QW, once per week; T_max,ss_, time to reach C_max_ at steady state; t_1/2_, elimination half-life; Vd_ss_/F, apparent volume of distribution after subcutaneous administration at steady state.

**Supplemental Table 3.** **Dose proportionality test on -normalized C_max_ and AUC_last_**

|  | **C_max_/Dose**  **((ng/mL)/(mg/kg))** | | **AUC_last_/Dose**  **((h*ng/mL)/(mg/kg))** | |
| --- | --- | --- | --- | --- |
|  | **Single dose** | **Multiple dose** | **Single dose** | **Multiple dose** |
| **Cohort 1 (n=5)** | 301.52 (363.95) | 785.00 (923.74) | 31051.12 (35518.71) | 77975.58 (82928.66) |
| **Cohort 2 (n=5)** | 2148.00 (975.34) | 6212.67 (3243.45) | 195413.60 (78119.08) | 721204.70 (401178.36) |
| **Cohort 3 (n=5)** | 2292.12 (1232.32) | 8339.39 (3855.84) | 221971.92 (118286.41) | 1142332.90 (574420.71) |
| ***P* value^*^** | .0092 | .0087 | .0133 | .0060 |
| **β1 (90% CI)^†^** | 6.81 (4.76, 8.85) | 6.71 (5.08, 8.34) | 6.32 (4.49, 8.14) | 7.01 (5.44, 8.58) |

^*^*P* value calculated from Kruskal-Wallis test. **^†^**Slope (β1) and 90% confidence interval (CI) from the power model. AUC_last_, area under the plasma concentration-time curve from the time of dosing to the time of the last measurable concentration; C_max_, maximum plasma drug concentration.

**Supplemental Figure 1.** **Dose proportionality assessment of PD parameters with a power model after multiple dosing.** E_max_ curves of (A) free TFPI, (B) diluted PT, (C) peak thrombin generation, (D) d-dimer, (E) fibrinogen, and (F) prothrombin fragment 1+2. E_max_, maximum observed response; PT, prothrombin time; TFPI, tissue factor pathway inhibitor. The gray shaded area represents the 90% CI. The blue line represents the median.

**Supplemental Figure 2. Exploratory plotting of the percent change in free TFPI from baseline and MG1113 plasma concentration, overlaid with bleeding events.** Bleeding events were marked as red dots. The horizontal red line indicates a reduction to 25% of the baseline value, corresponding to 75% suppression of Free TFPI. The vertical blue line represents the MG1113 concentration of 300 ng/mL, which is associated with a reduced risk of bleeding.
